# Comparative Analysis of Data‐Driven Rescoring Platforms for Improved Peptide Identification in HeLa Digest Samples

**DOI:** 10.1002/pmic.202400225

**Published:** 2025-02-02

**Authors:** Jesus D. Castaño, Francis Beaudry

**Affiliations:** ^1^ Département de Biomédecine Vétérinaire, Faculté de Médecine Vétérinaire Université de Montréal Saint‐Hyacinthe Canada; ^2^ Centre de recherche sur le cerveau et l'apprentissage (CIRCA) Université de Montréal Saint‐Hyacinthe Canada

**Keywords:** fragment ion intensity prediction, mass spectrometry, peptide spectrum matches (PSM), rescoring functions, search engines

## Abstract

Mass spectrometry is a critical tool to understand complex changes in biological processes. Despite significant advances in search engine technology, many spectra remain unassigned. This research evaluates the performance of three rescoring platforms, Oktoberfest, MS^2^Rescore, and inSPIRE, using MaxQuant output. The results indicated a substantial increase in identifications at the peptide level (40%–53%) and PSM level (64%–67%). However, some peptides were lost due to limitations in processing posttranslational modifications (PTMs)—with up to 75% of lost peptides exhibiting PTMs. Each platform displayed distinct strengths and weaknesses. For instance, inSPIRE performed best in terms of peptide identifications and unique peptides, while MS^2^Rescore performed better for PSMs at higher FDR values. Differences in platform performance stemmed from different sources: original search engine feature selection, type of ion series predicted, retention time predictor, and PTMs compatibility. Overall, inSPIRE showed a superior ability to harness original search engine results. Taken all together, rescoring platforms clearly outperformed original search results; however, they demanded additional computation time (up to 77%) and manual adjustments. The findings here underline the necessity of integrating rescoring platforms into current proteomics pipelines but also address some challenges in their implementation and optimization. Future integrated platforms may help enhance adoption.

## 1 Introduction

Mass spectrometry (MS)‐based proteomics is an essential tool for the identification of proteins in different biological samples. Proteins are important effectors in DNA maintenance, translation, cell cycle control, metabolite production, and other processes [1]. Thus, by comparing changes in protein expression under different conditions, it becomes possible to unravel the molecular basis of a variety of cellular processes. The use of proteomics techniques is particularly relevant due to their ability to gather proteome‐level information in a high‐throughput manner, which has sparked progress in areas such as healthcare [2, 3, 4], bioremediation [5], biomass degradation [6], or agriculture [7]—where proteins can be critical for successful applications. For instance, in biomedical sciences, it can lead to the identification of disease biomarkers, the development of targeted therapies, and a deeper insight into the mechanisms of diseases such as cancer [8], neurodegenerative disorders [9], and infectious diseases [10].

Comprehensive and accurate peptide identification, followed by protein inference, is at the center of MS‐based proteomics. This has substantially increased over the past decades due to the development of search engines that compare experimentally generated spectra with theoretical spectra from proteome databases [11]. Nonetheless, large portions of the gathered spectra are estimated to be unsuccessfully assigned [12]. While there are factors affecting peptide identification that are entirely dependent on sample preparation or instrument features, such as highly abundant proteins, peptide ionization efficiency, sequencing speed, or bias toward bigger proteins (>10 kDa) [13], there are other bioinformatic aspects that can heavily influence the results. In this regard, there has been tremendous work in the development of search engines and postprocessing strategies for nontargeted proteomics in the last few decades.

Search engines have been developed applying different scoring approaches such as the use of binomial distributions, generating functions, or dot products [14, 15, 16]. Some of the most used search engines include Andromeda, SEQUEST, FragPipe, and Comet, which have been incorporated into different proteomics platforms. The performance of search engines is generally enhanced by statistical postprocessing techniques such as Percolator [17], Mokapot [18], or PeptideProphet [19] that filter the results for a desired false discovery rate (FDR). These algorithms typically use semi‐supervised learning approaches to rescore and rank the peptide spectrum matches (PSMs) reported by a search engine. A complex scoring system that takes into account additional features can improve the performance of a database search tool, even rectifying incorrect assignments sometimes [17]. However, using a decoy database to determine the FDR in proteomics has limitations, including biased decoy generation with artificial decoy sequences, which may not represent true peptide diversity, leading to inaccurate FDR estimation [20]. The decoy database approach can also result in over‐ or underestimation of FDR due to nonrepresentative complexity and PTM differences [20, 21]. Dependence on the decoy generation strategy introduces variability, and the increased search space inflates computational demands. Additionally, real biological variants and isoforms may not be accounted for, further complicating accurate FDR determination [22, 23].

Despite these advances, the separation of correct and incorrect assignments is still a challenge. This difficulty likely leads to the loss of many true PSMs due to intrinsic drawbacks in the algorithms and the features they use. Current algorithms rely on scoring systems and heuristic rules that may not fully capture the complexity of the spectra, including variations caused by PTMs, co‐eluting peptides, and noise. These algorithms might prioritize high confidence matches but inadvertently discard true PSMs that exhibit atypical or less common patterns. Additionally, the features used for scoring might not be sufficient to distinguish between correct and incorrect matches in all cases. The complexity of biological samples, with their dynamic range of protein concentrations and modifications, further exacerbates these limitations. As a result, the balance between sensitivity (identifying true positives) and specificity (avoiding false positives) is difficult to achieve, leading to a significant number of true PSMs being overlooked or misclassified. To improve identification rates, more sophisticated algorithms and advanced machine learning approaches that can better handle these complexities are needed.

Machine learning has provided some notable solutions to this problem with the development of algorithms that can accurately predict retention time (RT) and fragment ion intensities [24, 25, 26]. In typical workflows, experimental spectra are compared with unit‐intensity theoretical spectra and then filtered by Percolator or other postprocessing techniques. By adding an intensity feature to the identification of fragment ion peaks, peptide identification can be substantially improved [24], which makes predicted ion intensity one of the most important parameters to estimate the quality of PSMs. These findings have sparked the development of multiple neural network‐based platforms for peptide prediction, such as MS^2^Rescore [27], Oktoberfest [28], inSPIRE [29], INFERYS Rescoring [30], and MSBooster [31]. These platforms use what is known as data‐driven rescoring workflows, in which additional features are integrated into the initial search engine results.

By leveraging the power of machine learning algorithms, these platforms address many of the limitations associated with traditional scoring, resulting in more accurate and comprehensive peptide identification—which is crucial for understanding complex biological systems and diseases. However, INFERYS Rescoring is implemented only in the commercial software Proteome Discoverer, which limits its wider application. Similarly, MSBooster, although free for academic users, was primarily intended for implementation in the FragPipe workflow, which limits its versatility in terms of the type of output it can accept as a stand‐alone platform. On the other hand, MS^2^Rescore, inSPIRE, and Oktoberfest are open‐source software programs that can take the results from a number of search engines, making them an attractive option for a wider audience of proteomics users. In this study, we evaluated the performance of these three rescoring platforms using search database results from MaxQuant—a freely available software widely used by the proteomics community. The results here constitute a useful guide to explore the impact of data‐driven rescoring workflows in traditional proteomics experiments.

## 2 Materials and Methods

### 2.1 Peptide Separation and Tandem MS/MS

**FIGURE 1:**
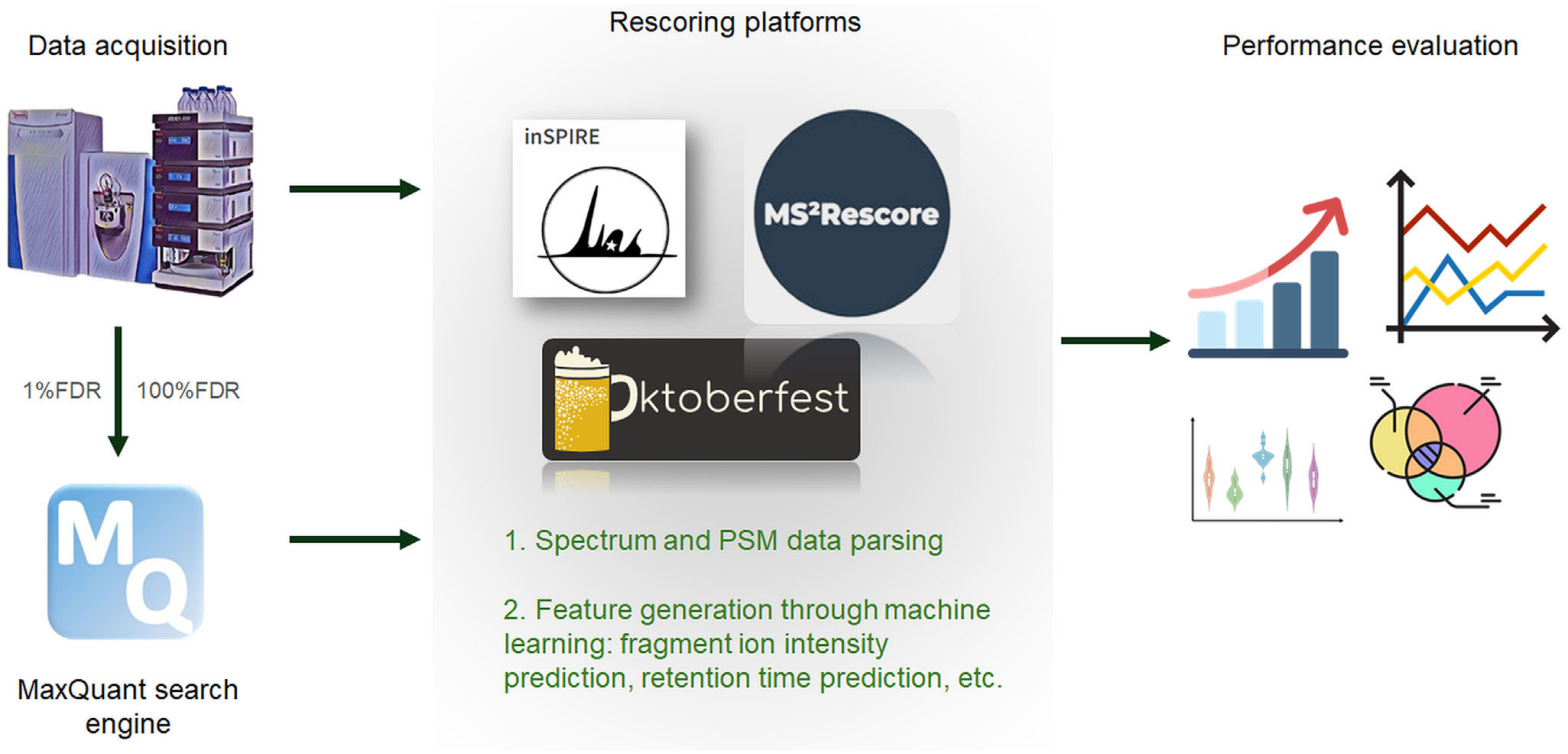
Overview of the methodology used in this study. Data acquisition in an Orbitrap mass spectrometer was followed by peptide search using MaxQuant. A 1% FDR was used to obtain the original results, and a 100% FDR was used for subsequent rescoring with inSPIRE, MS^2^Rescore, and Oktoberfest. The results were analyzed, and performance was compared against original MaxQuant results and between the rescoring platforms. FDR, false discovery rate.

A general overview of the workflow is presented in Figure 1. Standard HeLa protein digest samples (Thermo Fisher Scientific 88329) were dissolved in 0.1% formic acid following the manufacturer's instructions. The samples were analyzed in a Thermo Scientific Vanquish Neo UHPLC system (San Diego, CA, USA) with online chromatography separation in trap and elute mode. The trap column was a Thermo Scientific PepMap Neo 5 µm C18 300 µm × 5 mm, and the separation column was a Thermo Scientific PepMap Neo C18 2 µm × 75 µm × 500 mm. An amount of 1 µg of HeLa digest was used for the proteomics analysis. A 120‐min gradient was used from solvent A (0.1% formic acid in water) to solvent B (80% acetonitrile, 0.1% formic acid in water) at a flow rate of 200 nL/min. After the gradient, a 10‐min wash was applied, and the mobile phase composition was reestablished to its initial conditions over 10‐column volumes. MS/MS data acquisition was done by a Thermo Scientific Q Exactive Plus Orbitrap Mass Spectrometer, hooked up to a Nanospray Flex ion source (San Jose, CA, USA). Nanospray voltage was maintained at 2.2 kV in positive mode, with the transfer tube maintained at 200°C. Data collection was run on TOP 20 Data‐Dependent Acquisition (DDA) mode. The MS survey scan (350–1500 m/z) was performed at a resolution of 70,000 (full width at half maximum, FWHM) with an automatic gain control (AGC) set at 3 × 106 charges, and a maximum injection time of 100 ms. The top 20 most intense ions collected in MS1 (minimum intensity 10,000) were further fragmented with higher energy collisional dissociation (HCD) at 27 normalized collision energy (NCE). MS^2^ spectra were collected at a resolution of 17,500 (FWHM) and an AGC target value of 2 × 105 charges; the maximum injection time was 100 ms.

### 2.2 Database Searching and Output Analysis

Raw data was searched with the software MaxQuant 2.4.2.0, and the results processed with its accompanying tool Perseus 2.0.10.0 [32]. MaxQuant parameters were adjusted as follows: precursor tolerance (10 ppm); fragment tolerance (10 ppm); precursor charge (1–6); maximum peptide mass (3900 Da); peptide modifications (cysteine carbamidomethylation as fixed, and methionine oxidation and acetylation at N‐terminus, as variable). The database used was the Homo sapiens Proteome (UP000005640) reported on the Uniprot database, which contains a total of 82,678 entries. A reverse decoy database was used, and FDR for peptide‐spectrum matches (PSM) was set at 1% for the initial analysis. To exploit the capabilities of data‐driven rescoring functions, an initial run with 100% FDR was performed. Therefore, MaxQuant results at 100% FDR were used as input for the rescoring platforms. Data are available via ProteomeXchange with identifier PXD052875. Oktoberfest, inSPIRE, and MS^2^Rescore were used from the command line as indicated by the developers [27, 28, 29]. Details about the configuration files can be found in supplementary files S1 to S3. Acetylation was removed manually—using Microsoft Excel—from the output given by MaxQuant to run Oktoberfest and inSPIRE, since this modification is not currently supported by these platforms. Output files from the three rescoring platforms were analyzed manually to identify confidently identified peptides and PSMs.

### 2.3 Entrapment Database Search

To verify the accuracy of the rescoring platforms used here, we carried out an entrapment database method as described by others [33, 34]. Briefly, we developed an in‐house Python script to create entrapment sequences from the target proteins (for details, see supplementary files S4–S6). Target proteins were digested (trypsin), shuffled, and reassembled. Specifically, eight entrapment variants were created for every target protein, and the number of shuffle events was set at 10. The final list of entrapment variants was compared to the original target list to ensure all entrapment proteins were different from the original sequences. To evaluate the false match rate (FMR), we searched the raw data with MaxQuant as before but using the entrapment + target database. The FMR was calculated by Formula (1), where *N_trap_* represents the number of identifications at 1% FDR that matched the entrapment database and *N_target_*, the number of identifications that matched the target database:

(1) $$FMR\;\left(\%\right)=\frac{N_{trap}}{N_{target}}\;x100$$

## 3 Results and Discussions

**FIGURE 2:**
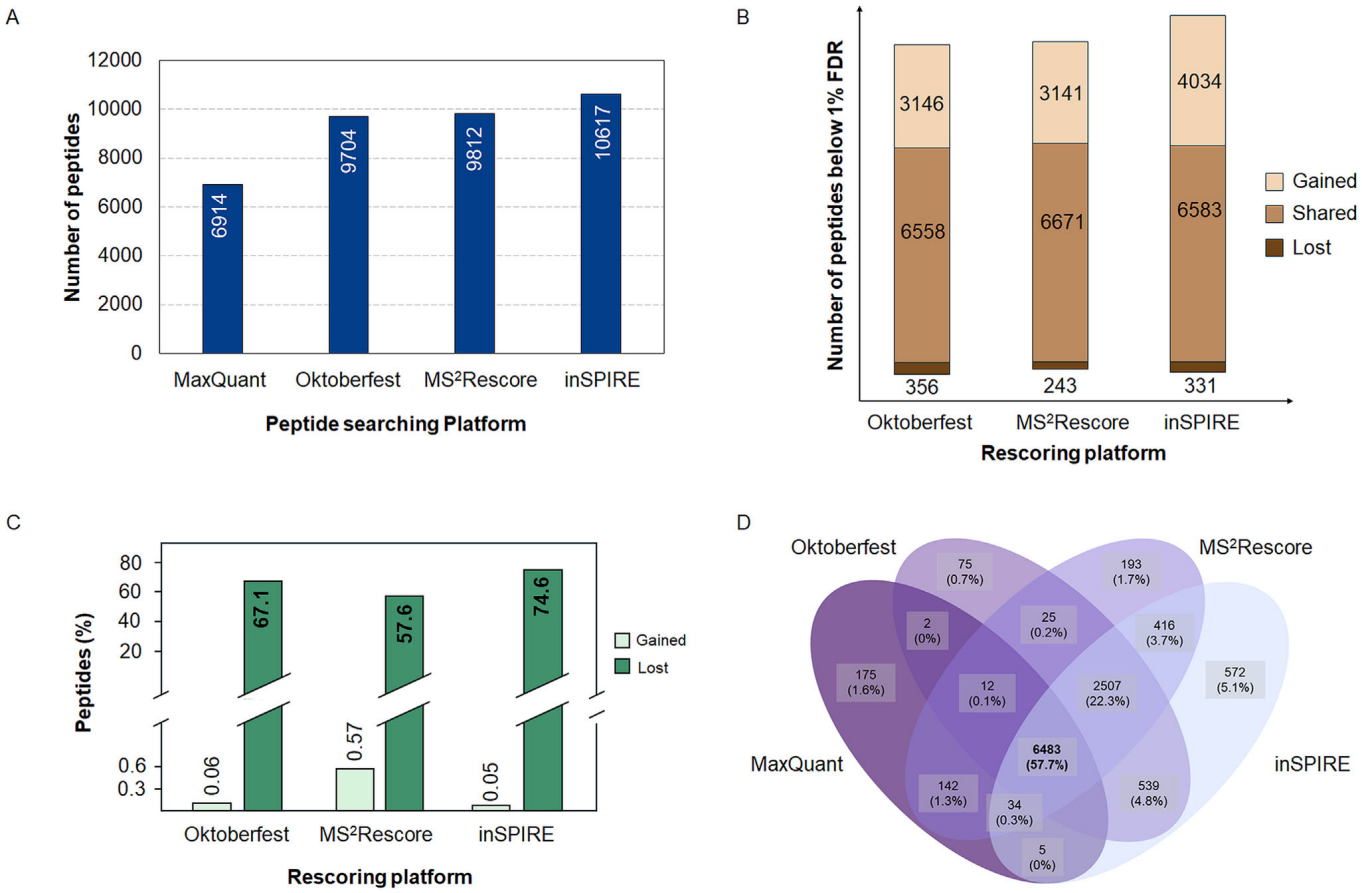
(A) Number of peptides identified using MaxQuant and the three rescoring platforms used (MaxQuant, Oktoberfest, and InSPIRE). (B) Peptides confidently identified (below 1% FD) that were gained, shared, or lost by the rescoring platforms compared to MaxQuant original results. (C) Proportion of modification containing peptides that were gained and lost by the rescoring platforms with respect to MaxQuant's original results. (D) Venn diagram showing the number of peptides shared among MaxQuant's original results and the three rescoring platforms evaluated.

The results clearly indicated an important increase (40%–53%) in the number of peptide identifications when using a data‐driven rescoring (Figure 2A) workflow. Importantly, the number of peptides confidently identified by the three rescoring platforms shared most of the peptides originally identified by MaxQuant while adding new identifications. Nonetheless, a small fraction of peptides was lost after the rescoring process (3%–5%) (Figure 2B). This was largely in part due to the inability of the rescoring platforms—especially Oktoberfest and inSPIRE—to process modifications other than fixed carbamidomethylation (C) and oxidation (M) [28, 29]. Indeed, most of the lost peptides contained modifications as shown in Figure 2C. At the same time, it was evident that only a very small fraction of the peptides identified by the rescoring engines contained modifications (<0.6%). Considering unmodified peptides only, the percentage of lost peptides dropped close to 1%, which would be expected given the 1% FDR used in the original results by MaxQuant.

Our findings clearly indicated a superior performance of inSPIRE for the analysis of HeLa tryptic samples, displaying over 500 peptides uniquely identified by this platform (Figure 2D). These results are in line with previous results where inSPIRE exhibited heightened performance, particularly for immunopeptides [29]. Although our assessment focused exclusively on the rescoring of MaxQuant results—since it was the only freely available search engine supported by all three platforms under scrutiny—it is crucial to recognize that search engine selection can deeply affect identification numbers [35, 36, 37]. Different search engines could significantly alter the total number of PSMs available for rescoring (100% FDR) as well as offer diverse features to be harnessed by the rescoring platform [29]. Therefore, the implementation of any pipeline using in‐silico spectral prediction rescoring should take this into consideration.

Considering that MS^2^ fragmentation patterns are highly dependent on the instrument and its corresponding settings (i.e., higher energy collision induced dissociation—HCD), it is important to mention that all the experimental data must come from the same instrument run under the same parameters. The first step, thus, is the calibration of the collision energy used for spectral prediction. This feature is available in inSPIRE and Oktoberfest, and it is a typical part of the pipeline during rescoring analysis. On the contrary, MS^2^Rescore generalizes across collision energy settings [27]. This feature could explain the lower performance observed for MS^2^Rescore. The MS^2^Rescore pipeline does not undergo a calibration step, and it has been shown to underperform when higher collision energies are used (>30). Here, the calibration step by Prosit in either inSPIRE or Oktoberfest yielded a CE of 34, which is not ideal for MS^2^Rescore. Indeed, reduction in both explained ion current and *y* and *b* ion series Pearson correlation has been identified as affected by higher collision energies [27]. Thus, collision energy should be carefully chosen when analyzing data; optimal values can also change from engine to engine [38], which makes this a crucial step of the workflow.

Interestingly, MS^2^Rescore can compensate for the loss of peptides caused by underperforming spectrum prediction with DeepLC RT features [27], capturing peptides that would otherwise be lost. Strikingly, DeepLC can accurately predict RT for unseen modified peptides by using an algorithm that considers atomic composition instead of amino acid encoding, thus allowing it to transfer knowledge gained at the atomic level to unseen modifications [39]. Generally, this allows MS^2^Rescore to increase identifications for peptides with lower precursor intensities, and consequently, lower quality spectra. This combined with MS^2^Rescore's ability to detect N‐acetylations and other modifications, could explain the still high proportion of unique identifications despite its lower performance compared to inSPIRE.

**FIGURE 3:**
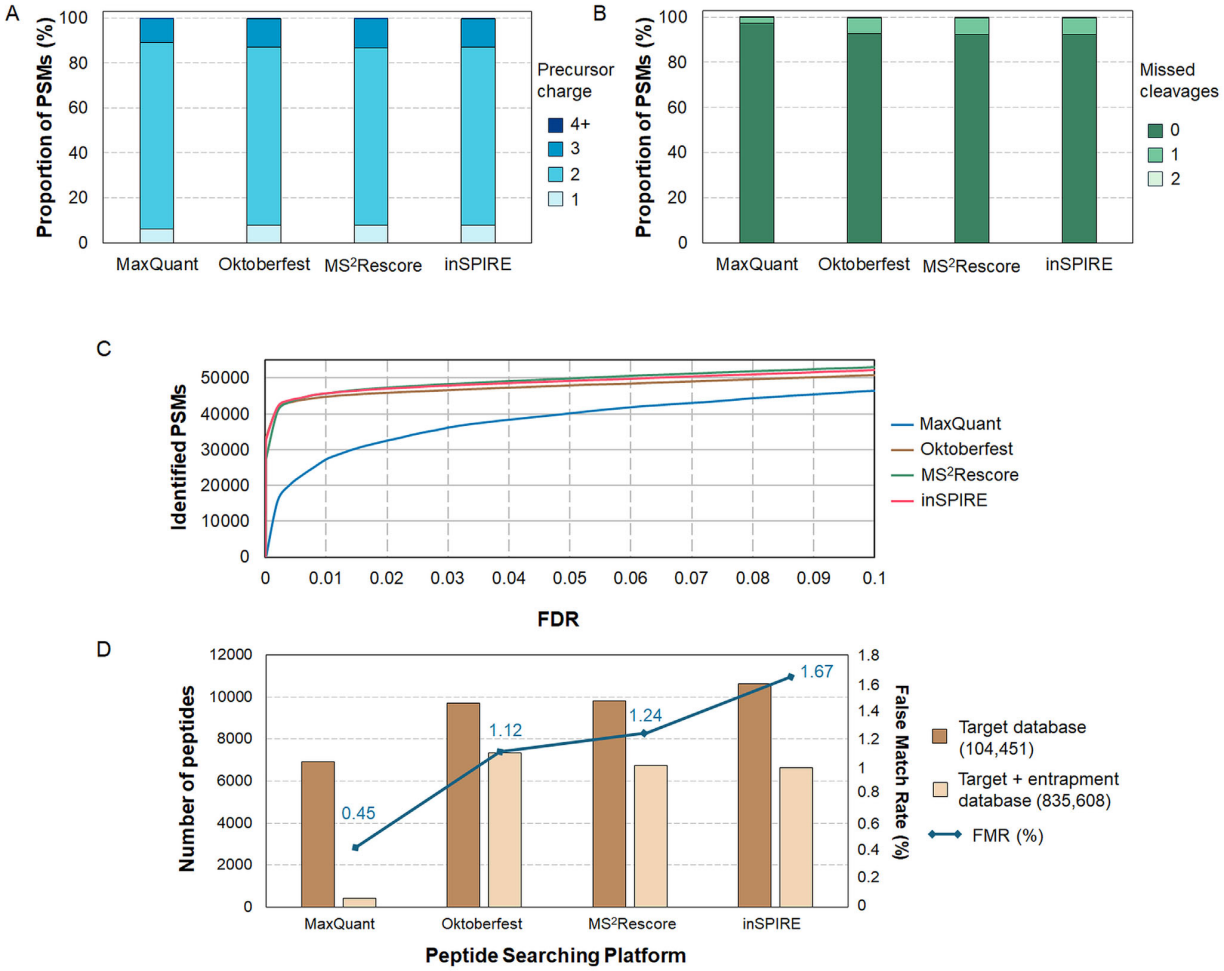
(A) Proportion of peptide spectrum matches (PSMs) displaying precursor charges between 1 and 4 identified by MaxQuant and each rescoring platform. (B) Proportion of PSMs containing up to two missed cleavages identified by MaxQuant and each rescoring platform. (C) Receiver operating curve (ROC) showing the number of identified PSMs as a function of the false discovery rate (FDR). (D) The analysis with the entrapment + target database shows that the number of wrong peptides identified (those from the entrapment database) represents a very low amount of the total peptides identified, with a false match rate (FMR) close to 1% (solid line). The target database results are included for comparison, highlighting the reduction in the number of peptide identifications when increasing the size of the database. The size of each database is included in parentheses.

When analyzing the results at the PSM level, we found that the number of PSMs identified by inSPIRE is higher below 1% FDR (Figure 3), which makes this platform particularly valuable for highly confident identifications. Above 1% FDR, the number of identifications by MS^2^Rescore reaches and surpasses that of inSPIRE—with both of these rescoring platforms outperforming Oktoberfest. Feature selection within each platform might be at the core of these differences. For instance, while MS^2^Rescore and Prosit rescoring pipelines focus on comparisons with relevant features to *b* and *y* ion series, inSPIRE also considers less dominant ion series. Even if tryptic peptide series are generally dominated by highly stable *y* ions, the addition of these features could provide additional resolution power for new identifications. This feature has been linked to the outstanding performance of this platform when working on immunopeptidomics, where the ion dominant series is often not the *y*‐series [27].

Another important difference that could explain the difference in performance between platforms is the number and weight of features considered by each platform. MS^2^Rescore reports displayed the use of 94 features, followed by Oktoberfest with 68, and inSPIRE with 34. While the number of rescoring features is higher with respect to MaxQuant's original results (14), it is possible that a higher number of features could trigger some overfitting when analyzing the same dataset. This could lead to a reduced number of PSMs and peptides meeting the Percolator threshold. In terms of weight, among the three rescoring platforms, fragment ion intensity prediction was the clear dominant feature that drove a higher number of identifications (Figure S1). Interestingly, the second most influential factor for inSPIRE included basic variables taken from the original data, such as sequence or delta score—compounded as MaxQuant in supplementary file S5—whereas RT prediction was the second most important feature for MS^2^Rescore and Oktoberfest. This demonstrates that beyond the use of fragment ion intensity predictions, the rescoring pipelines adopted for each platform could harness additional features from the original search engine results (at 100% FDR).

The enhanced results by the rescoring platforms also revealed differences in the proportion of precursor charge and missed cleavages. The rescoring platforms displayed a higher proportion of PSMs with precursor charges higher than 3 (∼13%) compared to MaxQuant original results (∼11%) (Figure 3A). In addition, a higher proportion of peptides with one or two missed cleavages was identified with rescoring platforms (>7%) compared to MaxQuant original results (<3%) (Figure 3B). Longer peptides with missed cleavages will contain *K* or *R* at the missed cleavage site, which helps explain the occurrence of peptides with additional charges. It is not clear what elements within the rescoring platforms would specifically allow the detection of more peptides with missed cleavages. Nonetheless, considering that these platforms have performed better in metaproteomics and immunopeptidomics [27, 28, 29], where search spaces are bigger, it makes sense that more peptides with missed cleavages are identified.

The accuracy of the database searching strategies was evaluated by using an entrapment method (Figure 3D). In this method, entrapment sequences—sequences known not to be present in the sample—are joined with the target database, and this new expanded database is used for peptide identification. The entrapment hits identified during peptide search can then be considered false positive identifications under a specific FDR. The results showed that the accuracy of the three rescoring platforms was close to the 1% expected, with Oktoberfest giving the best results (1.12% FMR). Additionally, the use of the entrapment database, which is 8‐fold larger than the target database, highlighted the ability of rescoring platforms to handle significantly bigger search spaces compared to the original search engine—even if with some significant losses as well. Interestingly, Oktoberfest was the most robust platform in this regard as it suffered the lowest loss of identifications (24%) compared to MS^2^Rescore (31%) and inSPIRE (37%).

**FIGURE 4:**
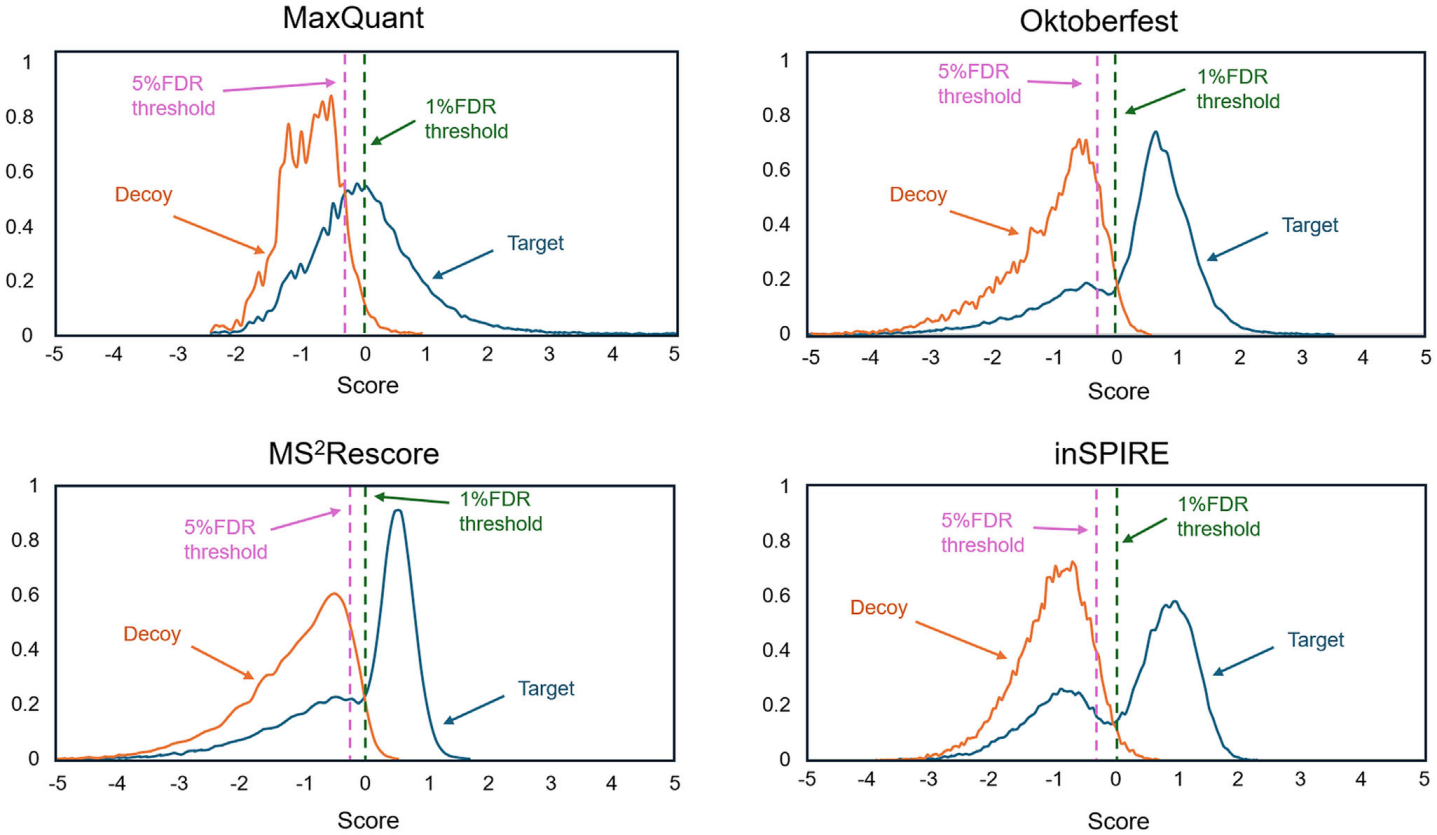
Density distributions for scores obtained for decoy and target peptides for MaxQuant original results and the three rescoring platforms evaluated. The dashed lines show where the 5% FDR and 1% FDR cut‐offs lie within the distributions. The area between the dashed lines for each distribution reflects the gain from using 5% FDR over 1% FDR at the cost of increasing possible false positives with a higher overlap with the decoy peptide distribution. FDR, false discovery rate.

Lastly, as stated before, the rescoring platforms substantially outperformed the original search engine results. The impact of the rescoring platforms can also be seen in terms of the density distributions for the scores of the decoy and target sequences for a given FDR (Figure 4). Enhancing the separation between density distributions for scores obtained from decoy and target peptides is critical in proteomics. This improvement boosts the ability to distinguish true matches from false positives, leading to more accurate identification of biologically relevant peptides and proteins. Clear differentiation between these distributions allows for more precise estimation of the FDR, ensuring reliable results and increased proteome coverage. After applying the rescoring pipelines, the overlap between decoy and target sequences is clearly reduced, which ultimately favored the identification of more PSMs and peptides. From the density distributions, it is also evident that while applying a 5% FDR to the original search engine results may yield a significant number of additional identifications, it also increases the risk of false positives overlapping with the decoy distribution. However, this issue is mitigated in the rescoring results, where the separation between true matches and false positives is improved, leading to more reliable identifications. Overall, these benefits should, however, be weighed against the need for additional computation time (50%–77%) (Figure S2), and manual manipulations of the search engine output to modify native search engine modification encoding. For instance, while it is true that MS^2^Rescore offered a higher number of peptide modifications, the equivalency of these modifications was not easy to map intuitively. Taken together, these drawbacks could lead to increased processing time, which could become prohibitive depending on the size of the data to process.

## 4 Conclusion

In conclusion, the use of rescoring platforms allowed a substantial increase of PSMs and peptide identifications for HeLa tryptic samples. At the present, the need for additional processing of output results and change of peptide modification encoding before rescoring could delay the wide implementation of these platforms. Also, the requirement to use command line programs could make it challenging for non‐expert users. However, we anticipate that future development of open‐source integrated platforms with friendly graphical interfaces will contribute to their wide adoption. In terms of performance, we found that all the rescoring platforms increased the identification of peptides containing missed cleavages, reflecting their increased capacity to confidently identify peptides within a bigger search space, which is a major challenge for current search engines. Finally, differences in performance among rescoring platforms could be attributed to the use of different features from the original search engine results, the recognition of different peptide PTMs, and the use of different algorithms for the prediction of fragment ion intensity and RT. Thus, the choice of the optimal rescoring platform could depend on various factors, which should be carefully considered depending on sample characteristics.

## Conflicts of Interest

The authors declare no conflicts of interest.

## Supporting information



Supporting Information

Supporting Information

Supporting Information

Supporting Information

Supporting Information

Supporting Information

Supporting Information

Supporting Information

Supporting Information

## Data Availability

The authors have nothing to report.
