# Supplementary material for: Comparative Analysis of Data‐Driven Rescoring Platforms for Improved Peptide Identification in HeLa Digest Samples
Source: Proteomics. 2025 Feb 2;25(7):e202400225. doi: 10.1002/pmic.202400225 (PMC11962579; doi:10.1002/pmic.202400225)
Supplement: Supplementary file 1 — Supporting Information [file PMIC-25-e202400225-s001.docx]

{

"type": "Rescoring",

"tag": "",

"output": "./out_oktoberfest_percolator_dual",

"inputs": {

"search_results": "./msms.txt",

"search_results_type": "Maxquant",

"spectra": "./mzML_files",

"spectra_type": "mzml"

},

"models": {

"intensity": "Prosit_2020_intensity_HCD",

"irt": "Prosit_2019_irt"

},

"prediction_server": "koina.wilhelmlab.org:443",

"numThreads": 1,

"fdr_estimation_method": "percolator",

"allFeatures": false,

"regressionMethod": "spline",

"ssl": true,

"thermoExe": "D:/ThermoRawFileParser/ThermoRawFileParser1.4.2/ThermoRawFileParser.exe",

"massTolerance": 20,

"unitMassTolerance": "ppm",

"ce_alignment_options": {

"ce_range": [19,50],

"use_ransac_model": false

}

}
