# Supplementary material for: Comparative Analysis of Data‐Driven Rescoring Platforms for Improved Peptide Identification in HeLa Digest Samples
Source: Proteomics. 2025 Feb 2;25(7):e202400225. doi: 10.1002/pmic.202400225 (PMC11962579; doi:10.1002/pmic.202400225)
Supplement: Supplementary file 2 — Supporting Information [file PMIC-25-e202400225-s005.docx]

{

"$schema": "./config_schema.json",

"ms2rescore": {

"feature_generators": {

"basic": {},

"ms2pip": {

"model": "HCD2021",

"ms2_tolerance": 0.02

},

"deeplc": {

"deeplc_retrain": false

},

"maxquant": {}

},

"rescoring_engine": {

"percolator": {

"write_weights": true,

"write_txt": true,

"write_flashlfq": false,

"protein_kwargs": {}

}

},

"config_file": "CLI_test_percolator-config.json",

"psm_file": [

"D:/Postdoc_UMontreal/34ADT-50cm-column/Top20-DDA/MaxQuant3_100FDR/Oktoberfest_Hela10/msms.txt"

],

"psm_file_type": "msms",

"psm_reader_kwargs": {},

"spectrum_path": "D:/Postdoc_UMontreal/34ADT-50cm-column/Top20-DDA/MaxQuant3_100FDR/Oktoberfest_Hela10/mzML_files",

"output_path": "D:/Postdoc_UMontreal/34ADT-50cm-column/Top20-DDA/MaxQuant3_100FDR/Oktoberfest_Hela10/HeLa_ms2rescore_out_percolator",

"log_level": "info",

"id_decoy_pattern": null,

"psm_id_pattern": null,

"spectrum_id_pattern": ".*scan=(\\d+)$",

"lower_score_is_better": false,

"modification_mapping": {

"Oxidation (M)": "U:Oxidation",

"Acetyl (Protein N-term)": "U:Acetyl"

},

"fixed_modifications": {

"C": [

"U:CARBAMIDOMETHYL"

]

},

"processes": 18,

"rename_to_usi": false,

"fasta_file": "D:/Postdoc_UMontreal/34ADT-50cm-column/Top20-DDA/MaxQuant3_100FDR/Oktoberfest_Hela10/uniprotkb_proteome_UP000005640_2023_08_22.fasta",

"write_report": true

}

}
