# Supplementary material for: Comparative Analysis of Data‐Driven Rescoring Platforms for Improved Peptide Identification in HeLa Digest Samples
Source: Proteomics. 2025 Feb 2;25(7):e202400225. doi: 10.1002/pmic.202400225 (PMC11962579; doi:10.1002/pmic.202400225)
Supplement: Supplementary file 3 — Supporting Information [file PMIC-25-e202400225-s006.docx]

---

experimentTitle: Example Rescoring

searchResults: msms.txt

searchEngine: maxquant

outputFolder: output_Inspire

scansFolder: mzML_files

scansFormat: mzML

deltaMethod: ignore

rescoreMethod: percolator

fixedModifications:

- Carbamidomethylation

mzAccuracy: 0.02

mzUnits: Da

nCores: 14
