# Supplementary material for: Comparative Analysis of Data‐Driven Rescoring Platforms for Improved Peptide Identification in HeLa Digest Samples
Source: Proteomics. 2025 Feb 2;25(7):e202400225. doi: 10.1002/pmic.202400225 (PMC11962579; doi:10.1002/pmic.202400225)
Supplement: Supplementary file 4 — Supporting Information [file PMIC-25-e202400225-s008.docx]

import random

import re

from Bio import SeqIO

from collections import defaultdict

def digest_protein(sequence, enzyme='trypsin'):

"""Digest a protein sequence into peptides based on the specified enzyme."""

# For simplicity, let's assume the enzyme is trypsin which cuts after 'K' or 'R' (except at the end)

peptides = re.split(r'(?<=[KR])(?!P)', sequence) # Split after K or R not followed by P

return [peptide for peptide in peptides if peptide]

def shuffle_peptide(peptide, n_shuffles=10):

"""Shuffle a peptide sequence up to n_shuffles times to generate a unique sequence."""

peptide_list = list(peptide)

shuffled = set()

for _ in range(n_shuffles):

random.shuffle(peptide_list)

shuffled.add(''.join(peptide_list))

if len(shuffled) >= n_shuffles:

break

return shuffled

def generate_entrapment_proteins(protein, n_variants=1):

"""Generate entrapment proteins by digesting, shuffling peptides, and reassembling."""

peptides = digest_protein(protein)

entrapment_proteins = []

for _ in range(n_variants):

shuffled_peptides = [random.choice(list(shuffle_peptide(p))) for p in peptides]

random.shuffle(shuffled_peptides)

entrapment_proteins.append(''.join(shuffled_peptides))

return entrapment_proteins

def calculate_fdp(target_proteins, entrapment_proteins):

"""Calculate the False Discovery Proportion (FDP)."""

target_set = set(target_proteins)

entrapment_set = set(entrapment_proteins)

false_positives = len(entrapment_set & target_set)

total_identified = len(entrapment_set)

fdp = false_positives / total_identified if total_identified > 0 else 0

return fdp

def main(fasta_file, n_variants=1, n_shuffles=10):

# Load target proteins from FASTA file

target_proteins = []

with open(fasta_file, 'r') as handle:

for record in SeqIO.parse(handle, 'fasta'):

target_proteins.append(str(record.seq))

# Generate entrapment proteins

entrapment_proteins = []

for protein in target_proteins:

entrapment_proteins.extend(generate_entrapment_proteins(protein, n_variants=n_variants))

# Calculate the false discovery proportion (FDP)

fdp = calculate_fdp(target_proteins, entrapment_proteins)

# Save target and entrapment proteins to new FASTA files

with open('target_proteins.fasta', 'w') as f:

for i, protein in enumerate(target_proteins):

f.write(f'>target_{i}\n{protein}\n')

with open('entrapment_proteins.fasta', 'w') as f:

for i, protein in enumerate(entrapment_proteins):

f.write(f'>entrapment_{i}\n{protein}\n')

print(f"False Discovery Proportion (FDP): {fdp:.2f}")

if __name__ == "__main__":

import argparse

parser = argparse.ArgumentParser(description='Generate entrapment database and calculate FDP.')

parser.add_argument('fasta_file', type=str, help='Input FASTA file with target protein sequences.')

parser.add_argument('--n_variants', type=int, default=1, help='Number of entrapment variants per target protein.')

parser.add_argument('--n_shuffles', type=int, default=10, help='Number of shuffles per peptide.')

args = parser.parse_args()

main(args.fasta_file, n_variants=args.n_variants, n_shuffles=args.n_shuffles)
