# Supplementary material for: Comparative Analysis of Data‐Driven Rescoring Platforms for Improved Peptide Identification in HeLa Digest Samples
Source: Proteomics. 2025 Feb 2;25(7):e202400225. doi: 10.1002/pmic.202400225 (PMC11962579; doi:10.1002/pmic.202400225)
Supplement: Supplementary file 5 — Supporting Information [file PMIC-25-e202400225-s002.docx]

#!/usr/bin/env python3

import sys

from Bio import SeqIO

def merge_fasta(file1, file2, output_file):

records = []

# Read records from the first file

with open(file1, 'r') as f:

records.extend(SeqIO.parse(f, 'fasta'))

# Read records from the second file

with open(file2, 'r') as f:

records.extend(SeqIO.parse(f, 'fasta'))

# Write all records to the output file

with open(output_file, 'w') as f:

SeqIO.write(records, f, 'fasta')

def main():

if len(sys.argv) != 4:

print("Usage: merge_fasta.py <file1.fasta> <file2.fasta> <output.fasta>")

sys.exit(1)

file1 = sys.argv[1]

file2 = sys.argv[2]

output_file = sys.argv[3]

merge_fasta(file1, file2, output_file)

print(f"Files {file1} and {file2} have been merged into {output_file}.")

if __name__ == "__main__":

main()
