# Supplementary material for: Comparative Analysis of Data‐Driven Rescoring Platforms for Improved Peptide Identification in HeLa Digest Samples
Source: Proteomics. 2025 Feb 2;25(7):e202400225. doi: 10.1002/pmic.202400225 (PMC11962579; doi:10.1002/pmic.202400225)
Supplement: Supplementary file 6 — Supporting Information [file PMIC-25-e202400225-s004.docx]

**Supplementary file S6**

**How to use the entrapment database generator script**

1. Run the script with python on the command line as follows, specifying the number of variants (how many fake proteins will be produced per target database protein) with the parameter “n_variants”, and the number of shuffles with the parameter “n_shuffles”. The protein database used a starting point to generate the entrapment database is called “my_proteins.fasta” in the example below.

python entrapment_database_generator.py my_proteins.fasta --n_variants 5 --n_shuffles 10

The script will generate two new files in the working directory: target_proteins.fasta and entrapment_proteins.fasta. Additionally, it will print the False Discovery Proportion (FDP) as well to make sure all the entrapment sequences are different from the target sequences.

1. Merge the two databases (entrapment + target) by using the merge_fasta_db.py script as follows:

python merge_fasta_db.py target_proteins.fasta entrapment_proteins.fasta output.fasta
