# Supplementary figures and images for: Comparative Analysis of Data‐Driven Rescoring Platforms for Improved Peptide Identification in HeLa Digest Samples
Source: Proteomics. 2025 Feb 2;25(7):e202400225. doi: 10.1002/pmic.202400225 (PMC11962579; doi:10.1002/pmic.202400225)

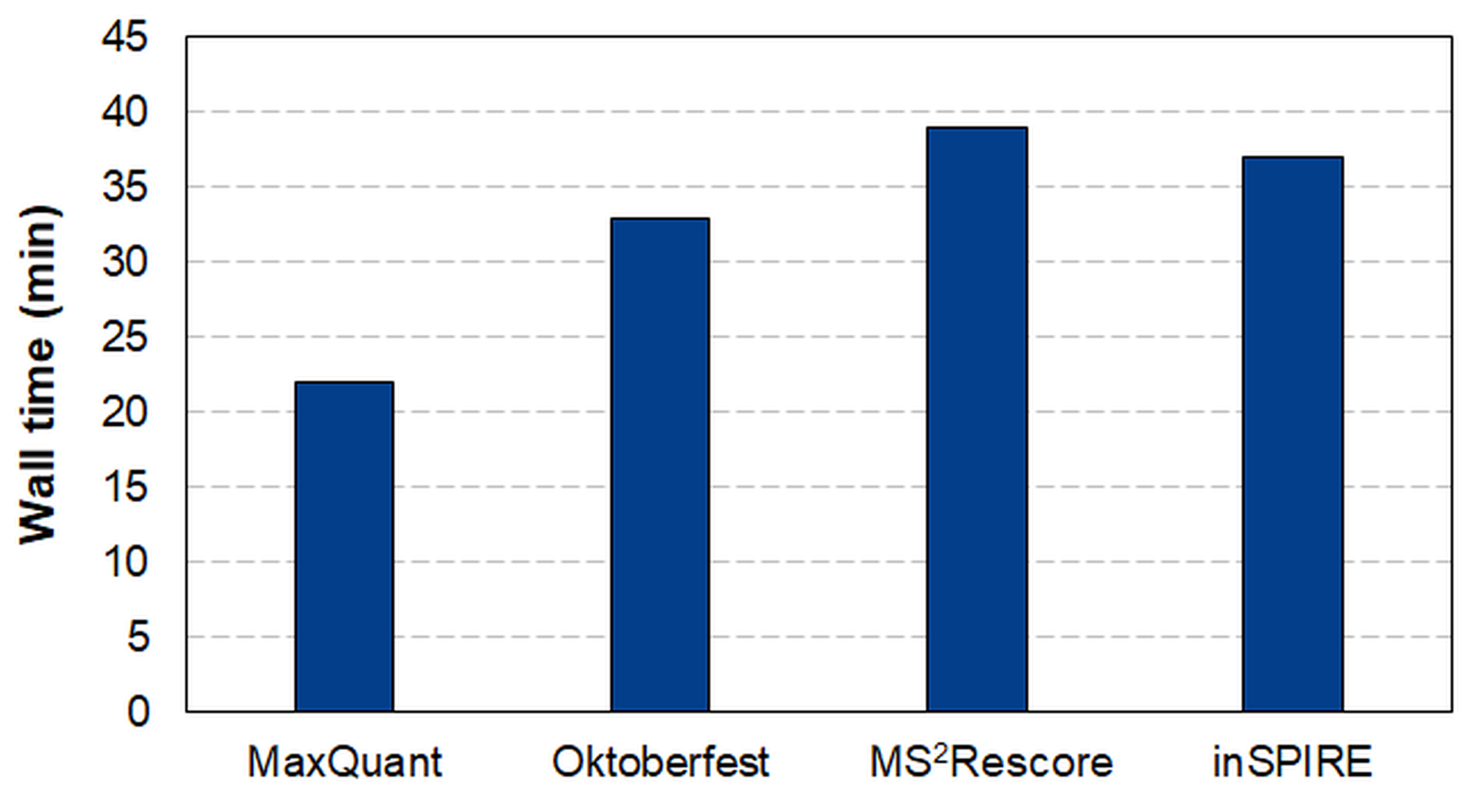

Supplement: Supplementary file 9 — Supporting Information [file PMIC-25-e202400225-s009.tif]
